# Prevention of pancreatic cancer in a hamster model by cAMP decrease

**DOI:** 10.18632/oncotarget.9790

**Published:** 2016-06-02

**Authors:** Jheelam Banerjee, Arokya M.S. Papu John, Mohammed H. Al-Wadei, Hildegard M. Schuller

**Affiliations:** ^1^ Experimental Oncology Laboratory, Department of Biomedical & Diagnostic Sciences, College of Veterinary Medicine, University of Tennessee, Knoxville, TN, USA

**Keywords:** pancreatic cancer, prevention, γ-aminobutyric acid, cyclic adenosine monophosphate, cancer stem cell signaling

## Abstract

Smoking and alcoholism are risk factors for the development of pancreatitis-associated pancreatic ductal adenocarcinoma (PDAC). We have previously shown that these cancers overexpressed stress neurotransmitters and cyclic adenosine monophosphate (cAMP) while the inhibitory neurotransmitter γ-aminobutyric acid (GABA) was suppressed. Using a hamster model, the current study has tested the hypothesis that cAMP decrease by GABA supplementation in the drinking water prevents the development of pancreatitis-associated PDAC. Our data reveal strong preventive effects of GABA supplementation on the development of PDAC and pancreatic intraductal neoplasia (PanIN). ELISA assays and immunohistochemistry revealed significant decreases in the levels of cAMP and interleukin 6 accompanied by reductions in the expression of several cancer stem cell markers and phosphorylated signaling proteins, which stimulate cell proliferation, and migration in pancreatic exocrine cells of GABA treated animals. We conclude that cAMP decrease by GABA supplementation inhibits multiple cancer stimulating pathways in cancer stem cells, differentiated cancer cells and the immune system, identifying this approach as promising novel tool for the prevention of PDAC in individuals with a history of smoking and alcoholism.

## INTRODUCTION

Pancreatic ductal adenocarcinoma (PDAC) is the fourth leading cause of cancer deaths in developed countries with a mortality near 80% within one year of diagnosis and incidence rising [1, 2]. PDAC is often associated with pancreatitis while smoking and heavy alcohol consumption are documented risk factors for both diseases [3]. In addition, a prospective study in women has identified *in utero* exposure via maternal smoking as a significant risk factor for the development of PDAC in the adult offspring [4].

We have previously reported the development of pancreatitis-associated PDAC in Syrian golden hamster offspring whose mothers were treated during their pregnancy with a single injection of the tobacco-specific carcinogenic nitrosamine 4-methylnitrosamino-(3-pyridyl)-1-butanone (NNK) one day before delivery of the pups and ethanol in the drinking water from day 5 through the end of pregnancy [5]. The experimentally induced PDACs demonstrated elevated levels of intracellular cyclic adenosine monophosphate (cAMP) while additionally overexpressing the α7 nicotinic acetylcholine receptor (α7nAChR) as well as vascular endothelial growth factor (VEGF), epidermal growth factor (EGF) and the phosphorylated signaling proteins ERK and CREB [6, 7]. By contrast, the levels of the inhibitory neurotransmitter γ-aminobutyric acid (GABA) and the expression of its synthesizing enzymes GAD65 and GAD67 were suppressed [7].

*In vitro* investigations have shown that human PDAC cell lines and immortalized human pancreatic duct epithelial cells synthesize and release the stress neurotransmitters norepinephrine and epinephrine in response to treatment with the α7nAChR agonists acetylcholine, nicotine or NNK, thereby increasing intracellular cAMP downstream of beta-adrenergic receptors (β-ARs), resulting in the phosphorylation of ERK, Src, AKT and CREB which increase proliferation and migration of human pancreatic cancer cells [8, 9]. In turn, gene knock-down of the α7nAChR inhibited the production of stress neurotransmitters and associated cell proliferation and migration [8–10]. Chronic *in vitro* exposure to nicotine additionally suppressed the synthesis and release of the inhibitory neurotransmitter γ-aminobutyric acid (GABA) by these cells whereas supplementation of GABA in the culture medium reversed the pro-proliferative and pro-migratory effects of nicotine via inhibition of cAMP formation by Gα_i_-coupled GABA-B-receptors [9]. In accord with these *in vitro* findings, human PDAC tissue micro-arrays expressed increased levels of norepinephrine and activated protein kinase A while GABA was suppressed. [11]. Investigations with mouse xenografts from human PDAC cell lines additionally showed that chronic psychological stress and the resulting systemic increase in stress neurotransmitters significantly promoted tumor growth via the cAMP-driven activation of multiple signaling pathways downstream of beta-adrenergic receptors while suppressing GABA [12]. Moreover, a recent study in an orthotopic mouse model of PDAC provided evidence that stress neurotransmitters released from sympathetic nerves in the pancreatic environment in response to chronic stress increased tumor progression and that this response was inhibited by beta-blocker treatment [13].

Collectively, these findings suggest that increased beta-adrenergic signaling caused by increases in stress neurotransmitter production at multiple anatomical sites and concomitant suppression of GABA in response to smoking or psychological stress has strong tumor promoting effects on PDAC and that blockade of this signaling cascade is a promising target for PDAC intervention. In accord with this interpretation, treatment with the general beta-blocker propranolol prevented the development of PDAC in our hamster model [6]. Epidemiological and clinical studies have additionally reported beta-blocker-induced improved clinical outcomes in several solid cancers [14–18], prompting the suggestion to utilize this family of cardiovascular drugs to improve responsiveness to current cancer therapeutic agents [19]. However, long-term treatment with beta-blockers can sensitize beta-adrenergic receptors [20, 21], a phenomenon that could potentially promote PDAC in individuals undergoing beta-blocker treatment for cancer prevention. This may be a reason, why negative effects of beta-blockers on pancreatic cancer outcomes have been reported [22].

The potential adverse effects of long-term beta-blocker therapy on pancreatic cancer can be circumvented by using a different approach that targets the effector cAMP downstream of beta-adrenergic receptors, leaving the upstream receptors unaltered. *In vitro* experiments in human PDAC cell lines have shown that reduction of cAMP formation by treatment with GABA had strong inhibitory effects on cell proliferation and migration induced by the beta-adrenergic receptor agonist isoproterenol [11] as well as nicotine [9] or ethanol [23]. Gene-knockdown of Gα_i_-coupled GABA-B receptors (GABA-B-Rs) blocked these effects of GABA while transient overexpression of GABA-B-Rs enhanced these responses to GABA, identifying the Gα_i_-mediated inhibition of adenylate cyclase activation as the underlying mechanism [11]. In accord with these *in vitro* findings, the progression of PDAC xenografts in athymic nude mice was significantly inhibited in the absence and presence of chronic exposure to nicotine or social stress by treatment of the animals with GABA in the drinking water [12, 24]. GABA treatment also enhanced the responsiveness of PDAC xenografts to the cancer therapeutic agent celecoxib in the absence and presence of social stress [25]. While these experiments have investigated the inhibitory effects of GABA on fully developed tumor cells, the current study has explored a potential cancer preventive effect of GABA in our hamster model of PDAC. Our data show that GABA supplementation had strong cancer preventive effects on pancreatitis-associated PDAC by decreasing cAMP and the pro-inflammatory cytokine interleukin 6 (IL-6), resulting in the simultaneous inhibition of multiple signaling pathways that stimulate the proliferation, progression and metastatic potential of differentiated PDAC cells and increase the self-renewal, clonogenicity and tumorigenicity of cancer stem cells of PDAC.

## RESULTS

**Figure 1:**
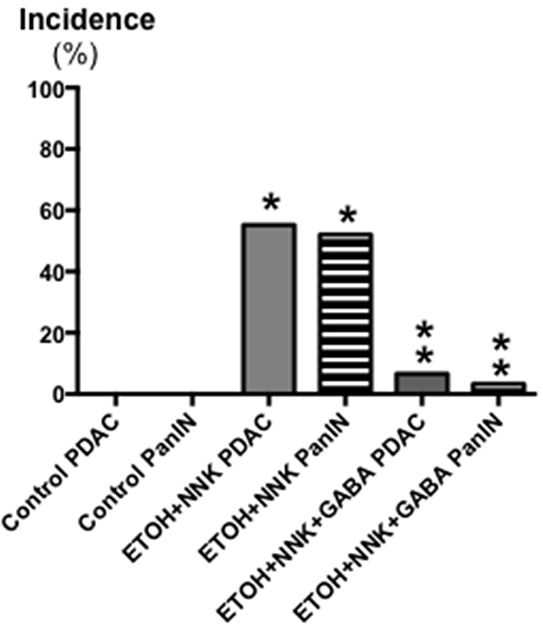
Effects of GABA supplementation on the incidence (%) of PDACs and PanINs in controls, hamsters treated prenatally with ETOH and NNK and in hamsters treated prenatally with ETOH and NNK followed by GABA supplementation in the drinking water at 4 weeks of age GABA had strong preventive effects on PDAC and PanIN. Significantly (p < 0.0001) different from controls (one asterix); significantly (p < 0.0001) different from group treated with ETOH and NNK alone (double asterix).

**Figure 2:**
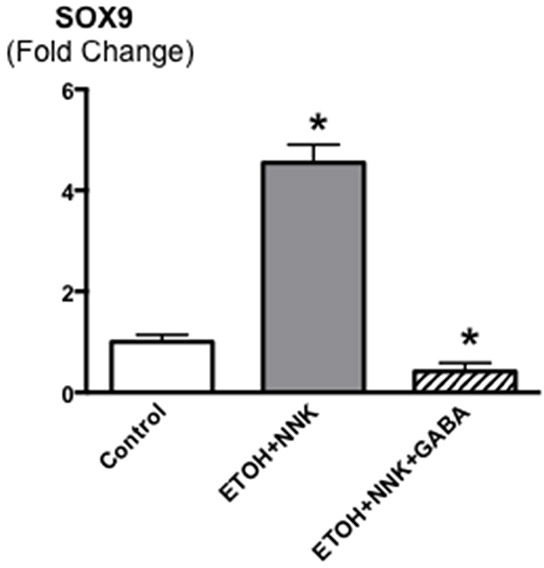
Levels of the transcription factor SOX9 that serves as a marker for pancreatic ductal lineage as assessed by ELISA assay in cells harvested by laser capture micro dissection from pancreatic ducts of control and GABA treated hamsters versus cells harvested from ETOH/NNK-induced PDACs SOX9 was strongly expressed in the tumor cells (control levels: 4.07 ± 0.41 ng/ml). Significantly (p < 0001) different from controls (asterix).

Control offspring had significantly (p < 0.0001) higher bodyweights throughout the experiment than the two groups whose mothers had been exposed to ethanol and NNK during pregnancy (data not shown) weighing 196g± 2.93 at the end of the study as opposed to 174.2g ±2.98 (ethanol + NNK) and 177.2g ±2.8 (ethanol + NNK followed by GABA supplementation). Figure 1 shows that 55.2% (16 animals; p < 0.0001) of the offspring whose mothers had been treated with ethanol and NNK developed PDAC and most of these animals had incidental pancreatic intraductal neoplasia (PanIN) classified as PanIN 3 based on the budding of cell clusters into the ductal lumen, often partially obstructing the duct (Figure 6A, 6B), a characteristic lacking in PanINs 1 and 2 [26–29]. Neither such preneoplastic lesions nor tumors were found in the pancreas of animals from the control group. GABA supplementation in the drinking water significantly (p < 0.0001) prevented the development of PDAC (6.7%; 2 animals) and PanINs (3.3 %; 1 animal). To assess the reproducibility of the cancer preventive effects of GABA, the fraction of animals with tumors or PanINs per each litter of hamsters in the treatment groups was analyzed by unpaired two-tailed t-test and showed significant (p < 0.001) inhibition of tumor and PanIN development in each GABA treated litter. ELISA assays showed expression of the marker for pancreatic ductal lineage, SOX9 [30], in the exocrine pancreatic cells and tumor cells harvested by laser capture micro-dissection (Figure 2). SOX-9 levels were significantly (p < 0.0001) higher in the tumor cells than in exocrine cells from controls or GABA treated hamsters (Figure 2).

**Figure 3:**
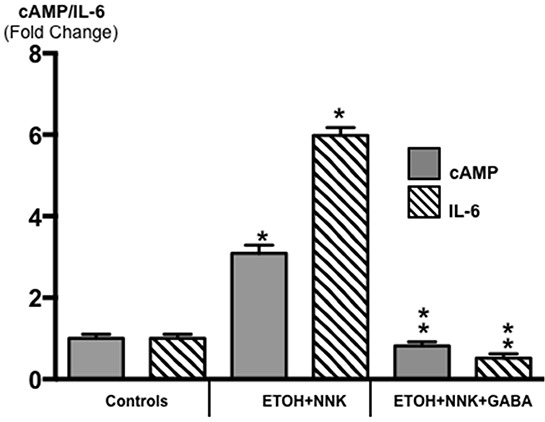
Modulation of intracellular cAMP and the pro-inflammatory cytokine IL-6 in exocrine pancreatic cells and tumor cells as assessed by ELISA assays PDAC cells (Tumor) in animals treated prenatally with ETOH plus NNK alone showed significant increases of cAMP (control levels: 5.1 ± 0.29 pmol/ml) and IL-6 (control levels: 10.81 ± 0.62 pg/ml) over the levels measured in exocrine pancreatic cells of controls. GABA supplementation completely reversed these responses, reducing cAMP and IL-6 in pancreatic exocrine cells below control levels. Significantly (p < 0001) different from controls (one asterix); significantly (p < 0.0001) different from tumor cells (double asterix).

The β-ARs are coupled to the stimulatory G-protein Gαs that initiates responses downstream of the receptors by activating adenylate cyclase, which catalyzes the formation of intracellular cAMP from adenosine triphosphate (ATP). In turn, increased levels of intracellular cAMP trigger the production and release of the pro-inflammatory cytokine IL-6 via transcriptional mechanisms [31–33]. IL-6 is over -expressed in most human pancreatic cancers and is synthesized and released by multiple cells in the tumor micro-environment, including tumor - associated macrophages, fibroblasts and pancreatic stellate cells. IL-6 also plays an important role in the development of pancreatitis [34]. Receptors coupled to the inhibitory G-protein Gαi function as physiological antagonists of β-ARs by inhibiting the activation of adenylate cyclase [35]. As Figure 3 shows, the levels of intracellular cAMP in the PDACs induced by ETOH+NNK were increased more than 3-fold compared with controls (p < 0.0001) while IL-6 was increased more than 6-fold (p < 0.0001.) By contrast, cAMP as well as IL-6 were suppressed below the levels in pancreatic tissues from controls (p <0.0001) in the animals treated with GABA supplementation.

**Figure 4:**
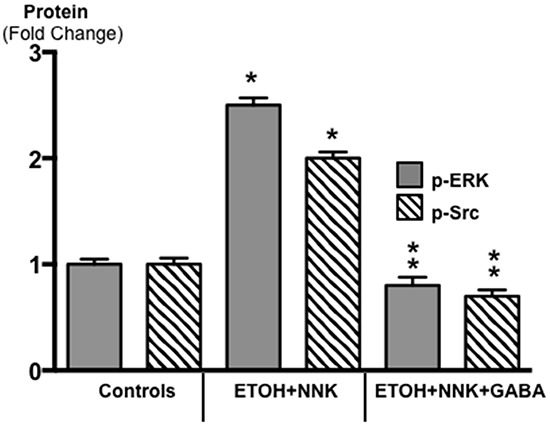
Results of ELISA assays for the determination of the phosphorylated forms of signaling proteins ERK and Src in micro-dissected PDAC cells induced by ETOH plus NNK and in exocrine pancreatic cells of controls and hamsters treated prenatally with ETOH plus NNK followed by GABA supplementation starting at 4 weeks of age The tumor cells showed elevated levels of p-ERK (control levels: 0.72 ± 0.5 ng/ml) and p-Src (control levels: 0.71 ± 0.07 ng/ml), responses reduced below control levels in exocrine pancreatic cells after GABA supplementation. Significantly (p < 0.0001) different from controls (on asterix); significantly (p < 0.0001) different from PDACs induced by ethanol and NNK (double asterix).

ELISA analyses of activated proteins that stimulate the proliferation and metastatic potential of tumor cells additionally showed significant increases in p-ERK (p <0.0001) and p-Src (p <0.0001) in the tumor tissues (Figure 4). GABA supplementation decreased the expression of both phosphorylated proteins below the levels observed in control animals (Figure 4; p < 0.0001).

**Figure 5:**
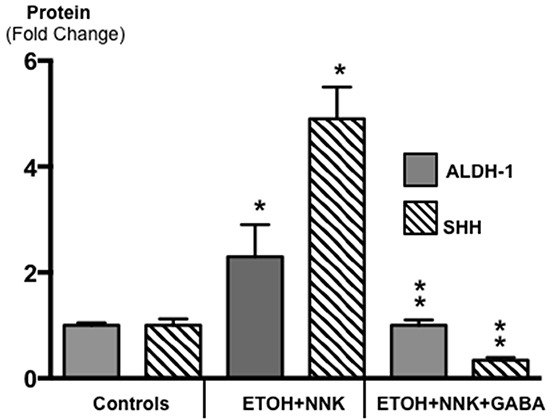
Results of ELISA assays for the determination of the cancer stem cell markers ALDH-1 and SHH in exocrine pancreatic cells and PDAC cells harvested by laser capture micro-dissection Both markers were significantly (p < 0.001) increased in PDACs induced by treatment with ETOH and NNK alone while GABA supplementation significantly (p < 0.0001) reduced both markers in exocrine pancreatic cells (Control SHH levels: 102.9 ± 6.1 pg/ml; control ALDH-1 levels: 2.03 ± 0.10 ng/ml). Significantly (p < 0.001) different from controls (one asterix); significantly (p < 0.0001) different fro group treated with ETOH + NNK alone (double asterix).

**Figure 6:**
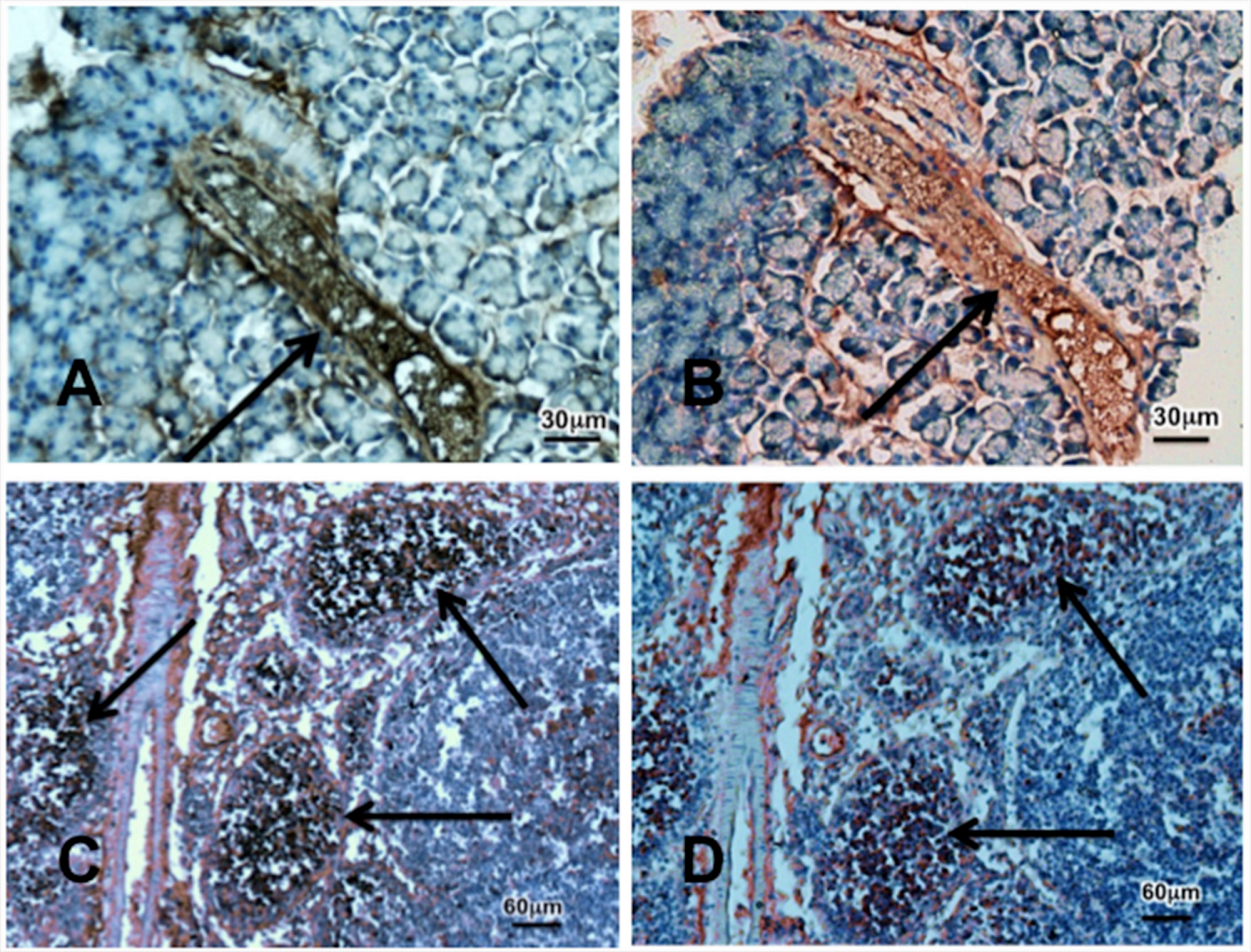
Photomicrographs exemplifying the over-expression of the cancer stem cell markers CD133 (brown stain) and Gli-1 (red stain) Photos A and B show a preneoplastic lesion PanIN-3 indicated by arrow in the pancreas of a hamster treated prenatally with ETOH and NNK while photos C and D show tumor tissue. Immunohistochemical stain was done with the Vectastain Elite ABC kit using incubation with exposure to primary anti-CD133 antibody (1:200) or Gli-1 (1:500) for 1 hour, DAB or AEC as substrate and hematoxylin as counterstain.

**Figure 7:**
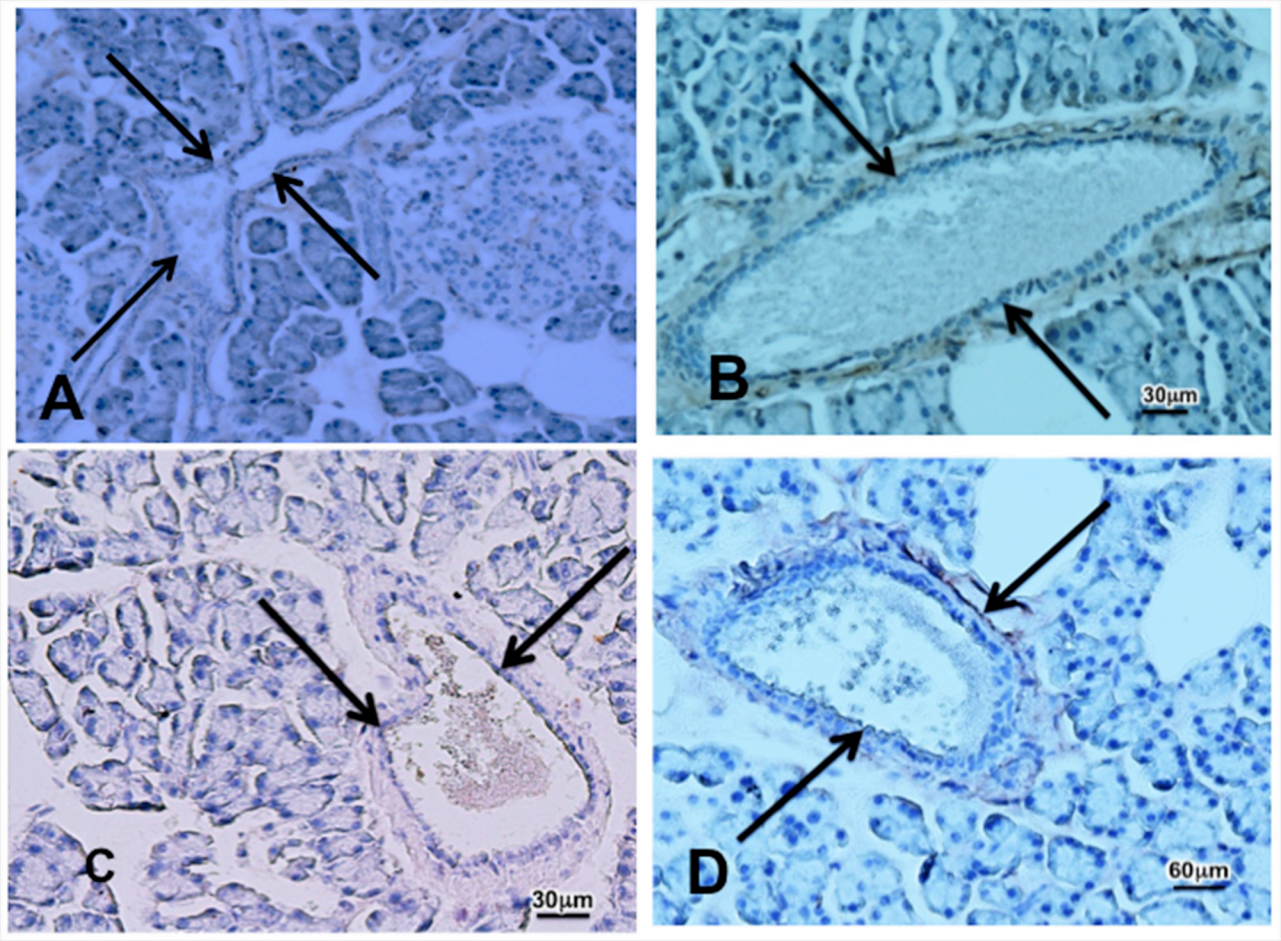
Photomicrographs showing faint immunoreactivity to the cancer stem cell marker CD133 **A.** In pancreatic tissue sections from a control (A) and from an animal that received cancer preventive treatment with GABA **B.** after prenatal treatment with ETOH and NNK and faint immunoreactivity for Gli-1 in a control **C.** and GABA treated **D.** animal. Pancreatic ducts are indicated by arrows.

**Figure 8:**
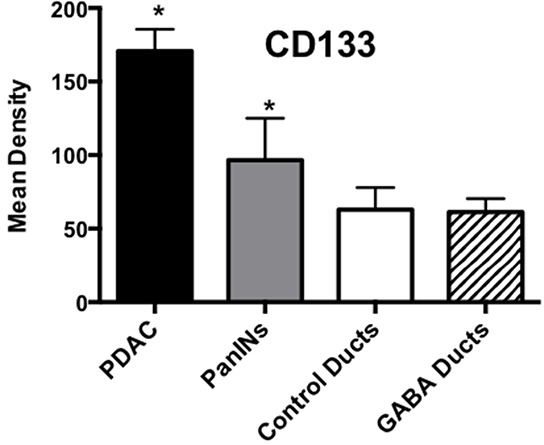
Results of densitometry for the quantitative assessment of CD133 immunoreactivity Tumor tissues and PanINs expressed significantly (p < 0.0001; asterix)) higher levels of CD133 immunoreactivity than duct epithelia in controls and GABA treated hamsters. Data are expressed as mean values and standard deviations at the 95% confidence interval from 50 mean density measurements per column.

**Figure 9:**
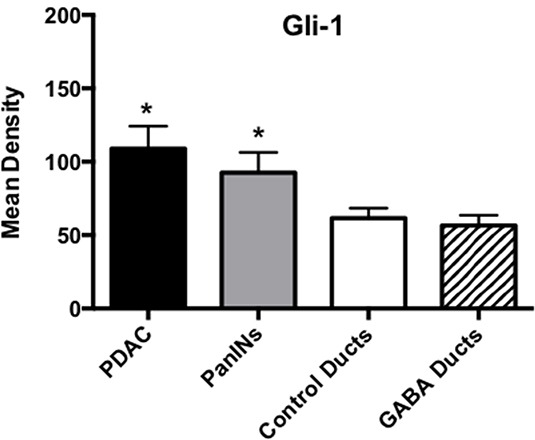
Results of densitometry for the quantitative determination of Gli-1 immunoreactivity Tumor tissues and PanINs expressed significantly (p < 0.0001; asterix)) higher levels of CD133 immunoreactivity than duct epithelia in controls and GABA treated hamsters. Data are expressed as mean values and standard deviations at the 95% confidence interval from 50 mean density measurements per column.

Cancer stem cells play important roles in cancer initiation and progression and the cancer stem cell markers aldehyde-1 dehydrogenase (ALDH-1) and sonic hedgehog (SHH) are therapeutic targets of pancreatic cancer [36–38]. In addition, pancreatic cancer and PanINs frequently express the cancer stem cell marker CD133 [39, 40]. We therefore analyzed the levels of SHH and ALDH-1 by ELISA assays and the expression of CD133 and the SHH effector Gli −1 [41] by immunohistochemistry. ALDH-1 (Figure 5; p < 0.001) and SHH (Figure 5; p <0.001) which stimulate the self-renewal and clonogenicity of cancer stem cells [42, 43] were significantly increased in the tumor cells. The expression of both stem cell markers was reduced by GABA supplementation below the levels in control hamsters (Figure 5; p<0.0001). Immunohistochemistry additionally revealed increased positive immuno-reactivity to the cancer stem cell markers CD133 and Gli −1 in PanINs and in PDACs (Figure 6–9). By contrast, exocrine  pancreatic cells in the controls (group 1) and in animals with GABA supplementation (group 3) showed significantly (p < 0.0001) lower levels of immunoreactivity to either cancer stem cell marker as assessed by densitometry (Figures 7–9).

## DISCUSSION

Our data show that GABA supplementation has strong cancer preventive effects in a hamster model of pancreatitis-associated PDAC induced by the combined prenatal exposure to alcohol and NNK. Smoking and alcoholism are both risk factors for pancreatitis and PDAC [44, 45] and pancreatitis from any etiology is an independent risk factor for pancreatic cancer [3]. In addition, a prospective study with 24 years follow-up has shown that maternal smoking increases the risk for the development of pancreatic cancer in the adult progeny [4]. The observed GABA-induced inhibition of PDAC and PanINs thought to represent preneoplastic lesions during the development of PDAC [46], may thus be of particular translational relevance for the prevention of smoking and drinking-associated PDAC. The additionally observed reduction in bodyweights in offspring prenatally exposed to ethanol and NNK that was not alleviated by GABA supplementation is likely attributable to the documented adverse effects of alcohol on fetal and postnatal development [47].

The results of our ELISA assays of cells harvested by laser capture micro-dissection in conjunction with immunohistochemical analysis of tissue sections indicate that the impressive cancer preventive effects of GABA were mediated by significant decreases in intracellular cAMP, an effect that suppressed IL-6 and simultaneously inhibited multiple pathways associated with the development and progression of PDAC. Most notable here is the fact that this involved inhibitory effects at the level of cancer stem cells, differentiated cancer cells as well as the immune system.

Cancer stem cells are thought to be the driving force of cancer initiation, progression, metastasis and drug resistance and are among current targets of pancreatic cancer therapy [48, 49]. The stem cell biomarkers CD133, ALDH-1 as well as SHH and its downstream effector Gli-1 are involved in cancer stem cell self-renewal, clonogenicity, tumorigenicity, metastasis and drug resistance of numerous solid cancers, including PDAC [36–38, 50]. Gli-1 additionally stimulates SOX9 transcription [51], which provides an explanation for the observed significant increase of SOX9 in tumor cells. The GABA-induced reduction in the expression of CD133, ALD-1, SHH, Gli-1 and SOX9 thus suggests significant inhibitory effects of GABA on cancer stem cells as contributing factors to the observed PDAC prevention in the current study.

Activation of he signaling kinases ERK and Src stimulates the proliferation and migration of PDAC cell lines predominantly comprised of differentiated cancer cells and both kinases are among current targets of pancreatic cancer therapy [52, 53]. *In vitro* studies from our laboratory have shown that GABA inhibits the nicotine and ethanol-induced phosphorylation of both signaling proteins in pancreatic duct epithelial cells and PDAC cell lines [8, 9, 23]. In accord with these findings, the expression levels of p-ERK and p-Src were significantly increased in PDAC cells harvested from animals whose mothers had been exposed to ethanol and NNK during pregnancy and the expression of both phosphorylated kinases was reduced below control levels in exocrine pancreatic cells by GABA supplementation of the offspring.

IL-6 is a pro-inflammatory cytokine that contributes to the severity of pancreatitis as well as the initiation and progression of PDAC [34, 54] and is primarily released by recruited immune cells, including tumor-associated macrophages [55]. The important role of this cytokine for PDAC development is reflected in the high levels of IL-6 detected in micro-dissected PDAC cells of the current experiment. By contrast, the animals treated with GABA supplementation showed IL-6 levels in exocrine pancreatic cells significantly below the levels measured in untreated controls. These findings suggest that GABA had strong inhibitory actions on immune cells associated with the development and progression of PDAC. This interpretation is supported by reports that GABA is an important physiological inhibitor of the immune system that reduces the production of inflammatory cytokines by macrophages [56, 57].

**Figure 10:**
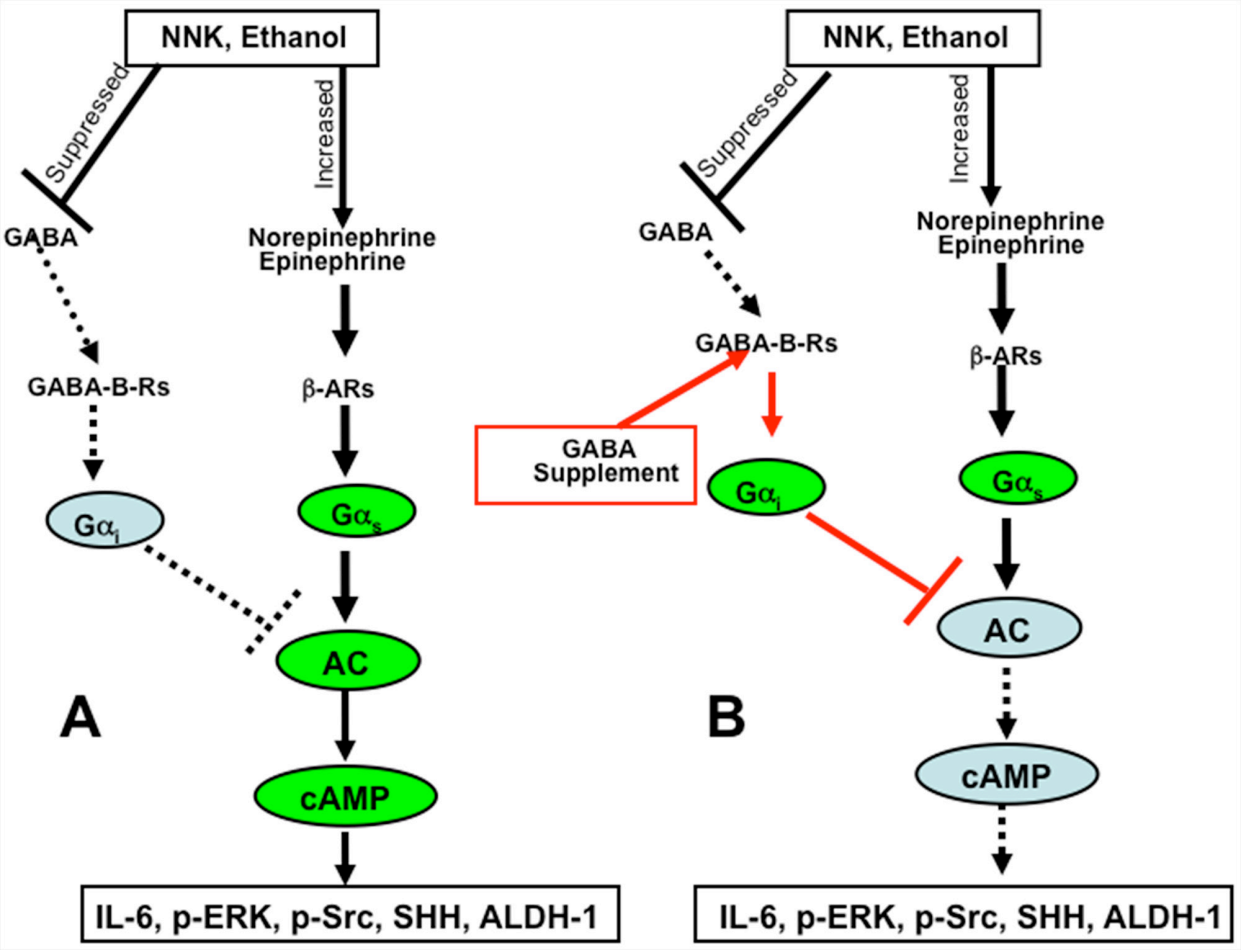
Cartoon illustrating the mechanisms of pancreatic cancer induction and progression induced by ethanol and NNK **A.** and the cancer preventive mechanisms of cAMP control by GABA supplementation **B.**

Our findings identify the restoration of suppressed GABA and resulting decrease of cAMP by GABA supplementation as an effective tool for the prevention of pancreatitis-associated PDAC in individuals at risk due to a history of smoking, alcoholism or prenatal exposure to either risk factor. Pancreatitis and PDAC both greatly reduce pancreatic GABA production because GABA producing endocrine and exocrine pancreatic cells are replaced by fibro-inflammatory tissues and cancer cells [58]. In addition, smoking increases the circulating levels of norepinephrine and epinephrine [59], both of which stimulate the proliferation and migration of PDAC cells and pancreatic duct epithelia via beta-adrenergic receptor signaling [8]. Collectively, these events shift the balance between PDAC stimulating and PDAC inhibiting neurotransmitters in such a way that cancer development and progression strive in a coordinated fashion at multiple levels, including cancer stem cells, differentiated cancer cells and the immune system (Figure 10). GABA has been used for many years as a nutritional supplement without adverse effects due to its calming and relaxing properties [60] and may be of use in individuals at risk for PDAC prevention as well as adjuvant therapy in PDAC patients whenever indicated by low systemic GABA levels and high stress neurotransmitter or cAMP levels. Epidemiological studies and clinical trials are now warranted to translate our preclinical findings to human pancreatic cancer.

## MATERIALS AND METHODS

The animal experiment was approved by the University of Tennessee Knoxville Institutional Animal Care and Use committee and all animals were maintained under the care of a veterinarian board certified in laboratory animal sciences in the AALAC approved laboratory animal facility of the Veterinary Medical Center. Nine timed-pregnant female Syrian golden hamsters (Charles River Laboratories, Wilmington, MA, USA) were housed one animal per cage in large cages (508mm X 254mm X 203mm) with free access to food (Purina Rodent Chow) and water and provided with nesting materials in accordance with guidelines of the American Association of Laboratory Animal Care. They were randomly assigned to three experimental groups; group 1: untreated control, group 2: treatment of pregnant females with ethanol and NNK with offspring receiving no further treatment, group 3: treatment of pregnant females with ethanol and NNK with offspring receiving cancer preventive treatment with GABA. As in previous experiments [5–7], exposure of the pregnant hamsters to ethanol was in the drinking water (10%) from day 5 to the end of pregnancy and treatment with NNK was by a single subcutaneous injection (50mg/kg in 0.2 ml sterile water) on day 15 of pregnancy. The total number of offspring for the three experimental groups was: 33 in group 1, 29 in group 2 and 30 in group 3. The pups were delivered on day 16 of pregnancy and remained undisturbed with their mothers until they were 4 weeks old to prevent cannibalism of the pups by the mothers. They were then weaned, and males and females of each litter were maintained separated by sex in identical large cages until fighting mandated the caging of the animals one hamster per cage (when the animals were about 6 months old). Cancer preventive GABA supplementation (10μM in the drinking water) in offspring of group 3 was started at 4 weeks of age and continued until the end of the study. All offspring were weighed weekly starting on the day of weaning and observed until one year of age at which time the animals were euthanized by CO_2_ inhalation. Full necropsies were performed and all major organs were fixed in 70% ethanol and embedded in paraffin. For routine histopathology evaluation, tissue sections were stained with hematoxylin and eosin. The incidence of pancreatic tumor versus no tumor and pancreatic intraepithelial neoplasia PanIN [26] versus no PanIN were tabulated in contingency tables and assessed for statistical significance between the three experimental groups by Chi-square tests, using Prism Graphpad software.

Pancreatic serial tissue sections of 5 micron thickness were additionally subjected to immunostains using Vectastain Elite ABC kits with 3-amino-9-ehtylcarbazole (AEC) peroxidase or 3,3′-diaminobenzidine (DAB) substrates and hematoxylin counterstain in accordance with the vendor's instructions (Vector Laboratories, Burlingame, CA, USA). Primary antibodies against the cancer stem cell marker CD133 (anti-CD 133 rabbit polyclonal, Biorbyt LLC, San Francisco, CA, USA; diluted 1:200 in PBS, exposure time: 1 hour) and the transcription factor Gli-1 (anti-Gli-1 rabbit polyclonal, Abcam, Cambridge, MA, USA; diluted 1:500, exposure time: 1 hour) that is activated by the sonic hedgehog pathway were used. Sections not exposed to primary antibody served as negative controls. Quantitative assessment of positive immunoreactivity was done by densitometry using NIH IMageJ for the determination of mean density of 50 random measurements per treatment group of duct epithelial cells, PanINs and tumor cells, respectively. Statistical analysis of these data was by non parametric Kruskal Wallis ANOVA and Mann Whitney tests.

Serial pancreatic tissue sections of 8micron thickness were used to harvest exocrine pancreatic cells and tumor cells by laser capture micro-dissection (PixCell IIe Laser Capture Micro-dissection system) following the vendor's instructions (Arcturus, Mountain View, CA, USA). Four aliquots of 10,000 cells each per treatment group were harvested and proteins were extracted using T-PER tissue protein extraction reagent (Thermo Fisher Scientific, Asheville, NC, USA). The proteins were then subjected to ELISA assays following instructions by the vendors for the detection of the transcription factor Sox-9 (Antibodies online, Atlanta, GA, USA), cAMP (Direct cAMP ELISA kit, Enzo Life Sciences, Farmingdale, NY, USA), the stem cell markers aldehyde dehydrogenase-1 (ALDH-1; ALDH-1 ELISA assay kit; Antibodies-Online Inc, Atlanta, GA, USA) and sonic hedgehog (SHH; SHH ELISA kit, Abcam, Cambridge, MA, USA), the phosphorylated signaling proteins p-ERK (Invitrogen, Carlsbad, CA, USA) and p-Src (MBL International Corporation, Woburn, MA, USA) and the pro-inflammatory cytokine interleukin-6 (IL-6; interleukin-6 mouse ELISA kit; Abcam). Statistical analysis of data generated with the ELISA assays was by one way ANOVA and unpaired two-tailed t-tests, using Prism Graphpad software.
